# Triplicate parallel life cycle divergence despite gene flow in periodical cicadas

**DOI:** 10.1038/s42003-018-0025-7

**Published:** 2018-04-19

**Authors:** Tomochika Fujisawa, Takuya Koyama, Satoshi Kakishima, John R. Cooley, Chris Simon, Jin Yoshimura, Teiji Sota

**Affiliations:** 1 Department of Zoology, Graduate School of Science, Kyoto University, Sakyo, Kyoto 606-8502 Japan; 2 Graduate School of Science and Technology, Shizuoka University, Hamamatsu, 432-8561 Japan; 3 Department of Botany, National Museum of Nature and Science, Tsukuba, 305-0005 Japan; 4 College of Integrative Sciences, Wesleyan University, Middletown, CT 06459 USA; 5 Department of Ecology and Evolutionary Biology, University of Connecticut, Storrs, CT 06268-3043 USA; 6 Department of Environmental and Forest Biology, State University of New York College of Environmental Science and Forestry, Syracuse, NY 13210 USA; 7 Marine Biosystems Research Center, Chiba University, Uchiura, Kamogawa, Chiba 299-5502 Japan

## Abstract

Periodical cicadas comprise three species groups containing three pairs of 13- and 17-year life cycle species showing parallel divergence, along with a more anciently diverged 13-year species (*Magicicda tredecim*). The mechanism and genetic basis of this parallel divergence is unknown. Here we use orthologous transcriptome sequences to explore the demographic processes and genomic evolution associated with parallel life cycle divergence. The three 13- and 17-year species pairs have similar demographic histories, and the two life cycles diverged 200,000–100,000 years ago. Interestingly, these life cycle differences have been maintained despite substantial gene flow between 13- and 17-year species within species groups, which is possible during co-emergences. Sequence divergence between 13- and 17-year species in each species group (excluding *M. tredecim*) is minimal, and we find no shared divergent single-nucleotide polymorphisms (SNPs) or loci associated with all instances of life cycle divergence. The two life cycles may be controlled by highly limited genomic differences.

## Abstract (web summary)

Tomochika Fujisawa et al. report transcriptome sequencing and demographic history analysis of periodical cicadas from three species groups within the genus *Magicicada*. They find evidence of gene flow between 13- and 17-year species despite the long-term maintenance of divergent life cycles, which may be controlled by unidentified genomic factors.

## Introduction

**Fig. 1:**
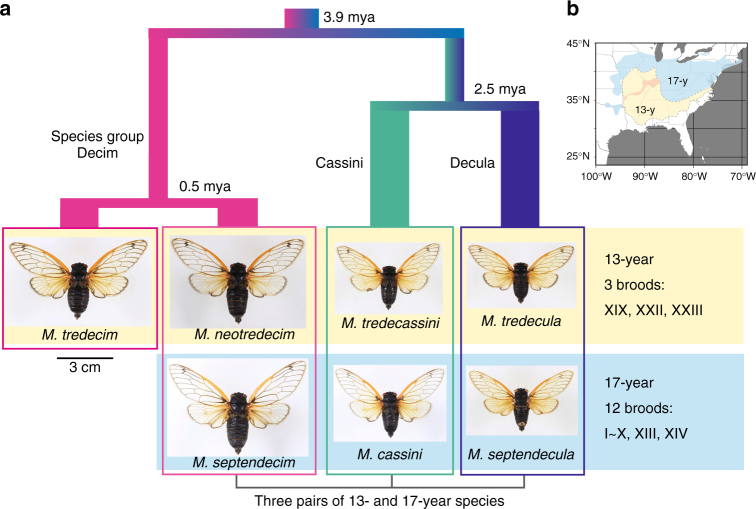
Species and species groups of *Magicicada* periodical cicadas. **a** Seven *Magicicada* species in three species groups, with relationships and divergence times for four lineages estimated in our previous study^6^. Individuals in photographs are all males. Photographs were taken by T. Sota. **b** Distribution ranges of 13- and 17-year cicadas. The dark pink area in the range of 13-year cicadas indicates the contact zone with *M. neotredecim* (northern species) and *M. tredecim* (southern species) in the Decim group^5^

Life history diversity is a remarkable feature of living organisms and underlies fundamental evolutionary questions^1^. Periodical cicadas of the genus *Magicicada* are found only in the eastern United States and are well known for their unusual life history patterns, characterised by prolonged juvenile periods of 13 or 17 years, followed by synchronised mass emergence of adults within local populations^2^. Only one cohort, or ‘brood’, of periodical cicadas emerges every 13 or 17 years in any given location. There are three co-occurring species groups of periodical cicadas, Decim, Decula and Cassini. Each has one species with a 17-year life cycle and one or two species with a 13-year cycle, and there are seven described species (four 13-year and three 17-year) in total^3–5^ (Fig. 1). Although the species groups clearly differ in morphology, male songs and female song preferences, the 13-year and 17-year species within each species group are extremely similar or indistinguishable in these characters^4,5^; thus, the difference in life cycle length is one of the only diagnostic characters for their identification.

The three species groups are estimated to have diverged 3.9–2.5 million years ago (mya), and subsequent divergence of the present 13-year (mostly southern) and 17-year (mostly northern) life cycles has occurred in parallel in the three species groups during the Quaternary, except for the first split of the 13-year species, *M. tredecim*, in the Decim group (0.5 mya) (Fig. 1)^6^. The synchronisation of prolonged life cycles among species groups is thought to have evolved for a predation-avoidance strategy^7^, an ecological problem shared among co-occurring species. The divergence of 13-year and 17-year life cycles may have been related to adaptation to climatic changes across glacial cycles; the 4-year extension of juvenile stages may have been advantageous for surviving in cooler northern environments^8,9^.

The genetic basis of life cycle length has not been studied because the long life cycles complicate genetic crosses. An early explanation for life cycle control in periodical cicadas proposed a one-locus, two-allele system in which either the 13- or the 17-year cycle is dominant^10,11^. Differences between the two life cycle lengths may be attributable to differences in juvenile developmental rate^12,13^, which may be regulated by one locus or a small number of loci. However, life cycle regulation in periodical cicadas may not always be strict, because 4-year acceleration and/or deceleration of emergences have been observed in both groups of cicadas, events unlikely to have resulted from fortuitous mass mutation^14^. These observations have led to the hypothesis that all periodical cicadas possess monomorphic developmental plasticity^14^ and that this common plasticity underlies the switching of life cycle lengths triggered by environmental cues (e.g., a drastic change in temperature during juvenile development), followed by a genetic change in a life-cycle control locus (genetic accommodation^15^), which enables a permanent life cycle shift^4^.

In general, parallelism in adaptive character divergence among closely related species results from parallel mutation or selection, ancestral polymorphism with balancing selection, or adaptive introgression^16,17^. In periodical cicadas, an ancestral polymorphism in life cycle length followed by collateral genetic evolution^16^ is considered the most parsimonious explanation for the parallel divergence and the formation of synchronous broods among three species groups, because multiple independent acquisitions of identical life cycles are unlikely^6^. In addition, a hypothesis of life cycle switching via introgressive hybridisation of the putative 13-year allele from 13- to 17-year cicadas has been proposed^10,11,18^. This hypothesis was used to explain the existence of two 13-year species in the Decim group^18^, proposing that introgressive hybridisation from the preexisting 13-year species *M. tredecim* to the 17-year *M. septendecim* produced the new 13-year species *M. neotredecim*. However, the hybrid origin hypothesis of *M. neotredecim* was rejected based on population genetic studies^5,19,20^. The hybrid origin hypothesis of 13-year species is unlikely to be applicable to the Cassini and Decula groups, which have no early diverged 13-year species (unless hybridisation between species groups drove life-cycle switching from 17- to 13-year cycles).

To understand the process and genetic basis of the parallel life cycle divergence observed in periodical cicadas, we inferred the demographic histories of broods of three *Magicicada* species groups. We used reduced representation sequences from transcriptomes (mRNA sequences) because *Magicicada* genomes are likely large in size (>6 Gbp) as in other cicadas^21^ and the whole genomes have not yet been sequenced. We focused on three pairs of 13- and 17-year species (excluding *M. tredecim*, which diverged earlier). In addition, we surveyed the genes responsible for life cycle control by comparing divergent loci of 13- and 17-year species pairs in the three species groups, which evolved in parallel. In general, comparisons of populations with parallel character divergence can be an effective means for discovering diverged portions of the genome and genes responsible for the character divergence^22,23^.

Our study reveals the historical process of the parallel life-cycle divergence in the three species groups. First we confirm the relationships of four major lineages (Cassini and Decula groups, and two lineages within Decim) and the absence of introgressive hybridisation among these four lineages. Then we estimate demographic histories in the three species groups and find that, in each group, 13-year broods are monophyletic, sister to or derived from 17-year broods. Interestingly, we find evidence of gene flow between the 13- and 17-year species in each species group. Finally we search for single-nucleotide polymorphisms (SNPs) or loci showing elevated divergence between life cycles, but do not find any divergent SNPs or loci shared among all 13- and 17-year species pairs, nor any evidence for parallel genomic divergence across all pairs. Thus, the genetic background of the life cycle divergence in periodical cicadas remains unclear.

## Results

### Assembly of transcriptome sequences and orthologous loci

**Fig. 2:**
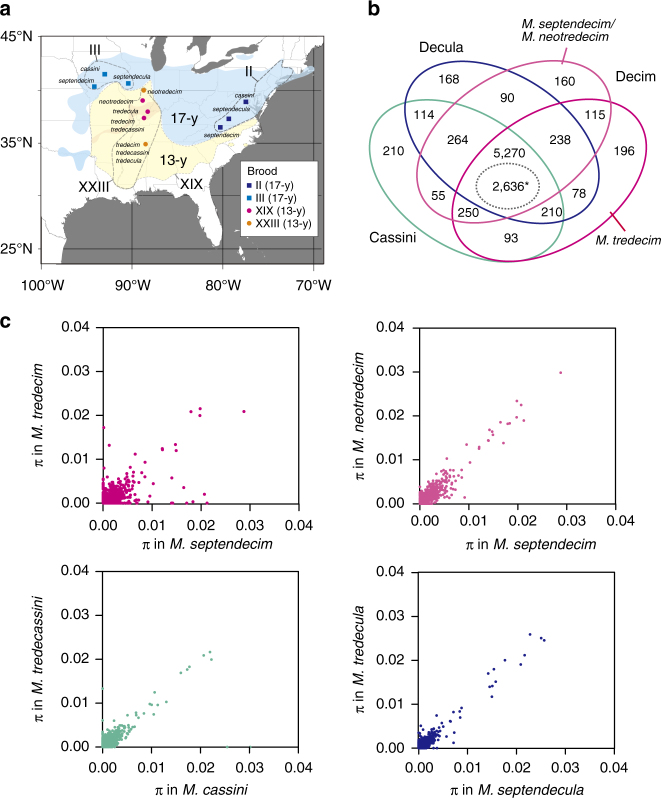
Sampling sites and the number and nucleotide diversity of orthologous loci. **a** Ranges of *Magicicada* broods used in this study (enclosed with broken lines) and locations of sampling sites (coloured dots). **b** Venn diagram for 7511 clusters (gene loci) orthologous to those of *Okanaga villosa* (outgroup). *A total of 2636 loci were used in the following analyses. **c** Nucleotide diversity values (*π*) of loci in seven species are plotted as correlation plots between 13- and 17-year species in each species groups. Note that *M. septendecim* (17-year) appears twice because there are two 13-year species in the Decim group. Median, mean and maximum values of *π* for each species: *M. tredecim*, median = 0.7×10^−4^, mean = 6.5×10^−4^, max = 0.2165; *M. septendecim*, median = 1.8×10^−4^, mean = 7.1×10^−4^, max = 0.02876; *M. neotredecim*, median = 1.3×10^−4^, mean = 6.9×10^−4^, max = 0.02984; *M. tredecassini*, median = 1.5×10^−4^, mean = 5.6×10^−4^, max = 0.02165; *M. cassini*, median = 1.2×10^−4^, mean = 5.6×10^−4^, max = 0.03015; *M. tredecula*, median = 0.0×10^−4^, mean = 5.3×10^−4^, max = 0.1865; *M. septendecula*, median = 0.0×10^−4^, mean = 4.5×10^−4^, max = 0.02567

We sequenced mRNA from head tissues of 28 individuals (Supplementary Data 1) from two representative 17-year broods (eastern and western broods, II and III, respectively) and the two major 13-year broods, XIX and XXIII (Fig. 2a). (Note that 12 broods with 17-year cycles and three broods with 13-year cycles currently exist). De novo assemblies of the RNAseq reads were generated separately for four distinct groups: Decim (*M. septendecim*, *M. neotredecim*); *M. tredecim*; Cassini (*M. cassini*, *M. tredecassini*); and Decula (*M. septendecula*, *M. tredecula*). For each group, we obtained 76,519–90,287 contigs (length: 300–26,405 bp) with an average N50 length of 1476 bp (Table 1). Using these contigs, 7511 clusters orthologous to contigs of the outgroup *Okanagana villosa* transcriptome sequence from Genbank were identified, of which 5270 were shared by all four *Magicicada* groups (Fig. 2b). Among the 5270 clusters, we identified 2636 clusters (orthologous loci) that contained data from *O. villosa* and at least 27 *Magicicada* samples for phylogenetic and demographic analyses. Of the 2636 loci, 99% had BLAST hits with e-values <1×10^–5^ in the RefSeq protein database (Supplementary Data 2). The average alignment length of the loci was 1627 bp, and the average nucleotide diversity (*π*) of the loci for all *Magicicada* sequences (*n* *=* 28) was 0.0019 (range: 0–0.0238). The nucleotide diversity of the loci within the seven species (*n* = 4 for each species) was generally low, with a mean of 0.00045–0.00071 and a median of 0–0.00018 (Fig. 2c).

**Table 1:** Assembly of sequence reads for four groups (lineages) of *Magicicada*

Species group	Species/broods^a^	*N* ^b^	No. contigs	Total contig length, bp	Max contig length, bp	Contig N50, bp	GC (%)	gap site (%)
Decim	*M. tredecim*/ XIX, XXIII	4	76,519	75,508,799	23,352	1511	34.5	0.0
Decim	*M. septendecim*/ II, III; *M. neotredecim*/ XIX, XXIII	8	90,287	87,384,502	25,741	1474	34.4	0.0
Cassini	*M. cassini*/ II, III; *M. tredecassini*/ XIX, XXIII	8	86,587	83,074,541	26,405	1441	34.4	0.0
Decula	*M. septendecula*/ II, III; *M. tredecula*/ XIX, XXIII	8	85,135	82,756,110	26,269	1478	34.5	0.0
Total		28	338,528	328,723,952				

Contigs shorter than 300 bp and short isoforms were excluded

^a^ Brood numbers are indicated by Roman numerals

^b^ Number of individuals used in the assembly. Two individuals per brood per species

### Molecular phylogeny of periodical cicadas

**Fig. 3:**
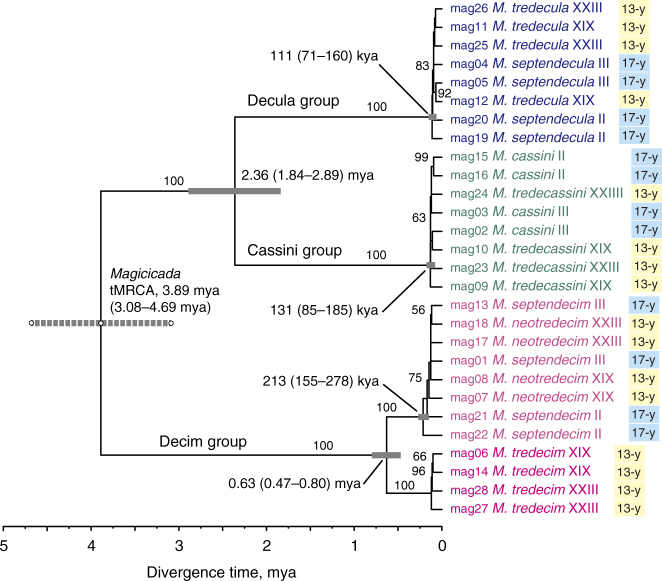
Maximum-likelihood (ML) tree resulting from the concatenated sequences for individual cicadas. The tree was made ultrametric with the time of most recent common ancestor (tMRCA) of all *Magicicada* set to 3.89 million years ago^6^ (the 95% confidence interval of this node is shown by the broken line). Numerals above the branches are the bootstrap percentages resulting from the ML analysis. Estimated ages and 95% confidence intervals based on two calibrations using the upper and lower confidence limits of *Magicicada* tMRCA are shown for five major nodes (grey bars)

**Fig. 4:**
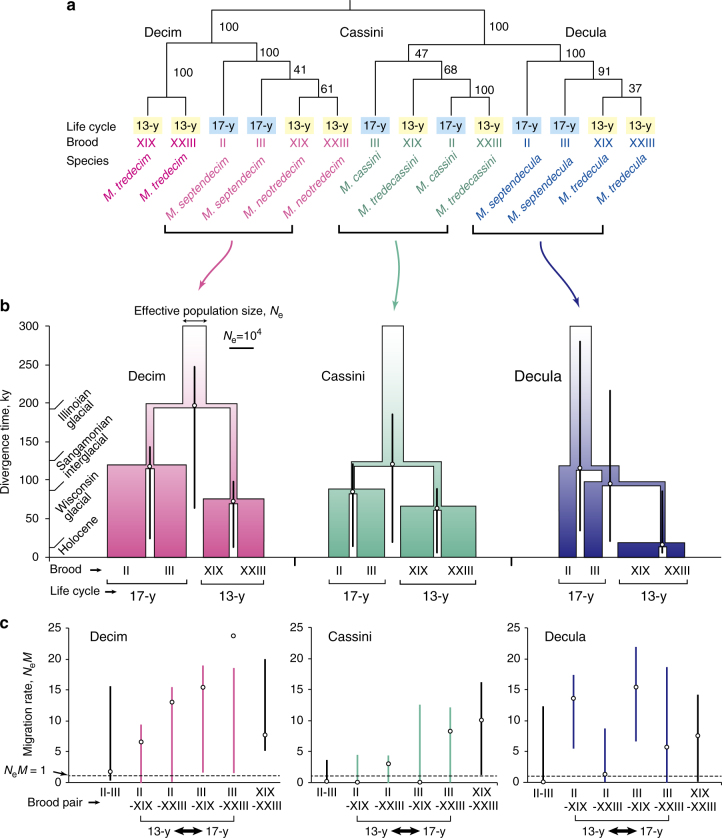
Relationship and demographic history of periodical cicada broods. **a** Phylogenetic relationship of broods reconstructed using SVDquartets. Numerals show bootstrap percentages of node recovery. **b** Demographic history of broods in each species group reconstructed using fastsimcoal2. Divergence of broods is shown with divergence times and effective population sizes (*N*_e_; width of column). Vertical bars for divergence times show 95% confidence intervals. Demographic parameters were scaled with the effective population size of one brood (brood XIX for Decim; brood II for Cassini and Decula). **c** Estimated gene flow (*N*_e_*M*) between broods (open circles). Solid lines show 95% confidence intervals

To characterise the historical relationship of species groups and broods, we first reconstructed phylogenetic trees using concatenated sequence data from the orthologous loci. The concatenated alignment was ca. 4.3 Mb in length, with 18% missing sites, and it contained 18,243 informative sites. The maximum-likelihood tree reliably recovered the monophyly of three species groups and the two lineages within the Decim group (*M. tredecim* and the lineage containing *M. neotredecim* and *M. septendecim*), but the relationships among broods within the Decim group (excluding *M. tredecim*), the Cassini group, and the Decula group were unresolved (Fig. 3). We also applied a species-tree method (SVDquartets^24^) to resolve the relationships among allochronically-separated broods, but it again poorly resolved the relationships among 13- and 17-year broods within each species group (Fig. 4a). In this tree, monophyly of the two 13-year broods was weakly supported in the Decim and Decula groups, whereas they were not monophyletic in the Cassini group.

### Lack of hybridisation between four major lineages

To reconstruct the process of life cycle divergence, we first tested whether introgressive hybridisation among the four lineages (i.e. *M. tredecim* and three paired 13-year and 17-year species) was involved in life-cycle divergence events using the ABBA-BABA test with the *D*-statistic^25,26^ for SNPs. In particular, we tested the possibility that the earliest-diverged 13-year species, *M. tredecim*, introduced the 13-year life cycle into another lineage of Decim, or the Cassini and Decular groups through hybridisation, but we found no evidence for introgressive hybridisation (Table 2). We also tested for hybridisation between *M. neotredecim* and the Cassini or Decula group, and between the Cassini and Decula groups, but found no positive evidence (Table 2). Thus, we excluded the possibility that introgressive hybridisation between species groups or between the distinct Decim lineages was involved in the life-cycle divergence process.

**Table 2:** Results of ABBA-BABA test with *D*-statistic for testing introgression hypotheses between 13-year species of different species groups or between 13-year species in the Decim group

Taxon assigned (species/brood)				No. of sites	*D* (STD^a^)	*Z* ^b^
P1	P2	P3	O			
(a) *M. tredecim* to *M. neotredecim*						
sdecim III	ntdecim XIX	tdecim XIX	cassini II,III	9225	−0.0048 (0.0701)	0.0680
sdecim III	ntdecim XXIII	tdecim XXIII	cassini II,III	7077	0.0053 (0.0773)	0.0690
sdecim II	ntdecim XIX	tdecim XIX	cassini II,III	9024	−0.0269 (0.0752)	0.3580
sdecim II	ntdecim XXIII	tdecim XXIII	cassini II,III	7037	0.0122 (0.080)	0.1526
(b) *M. tredecim* to *M. tredecassini*						
cassini III	tcassini XIX	tdecim XIX	*O. villosa*	336928	−0.0424 (0.0724)	0.5860
cassini III	tcassini XXIII	tdecim XXIII	*O. villosa*	280625	0.0369 (0.0779)	0.4741
cassini II	tcassini XIX	tdecim XIX	*O. villosa*	339643	−0.0616 (0.0752)	0.8193
cassini II	tcassini XXIII	tdecim XXIII	*O. villosa*	287058	0.0177 (0.080)	0.2209
(c) *M. tredecim* to *M. tredecula*						
sdecula III	tdecula XIX	tdecim XIX	*O. villosa*	346099	−0.027 (0.085)	0.3180
sdecula III	tdecula XXIII	tdecim XXIII	*O. villosa*	283106	0.0633 (0.0923)	0.6864
sdecula II	tdecula XIX	tdecim XIX	*O. villosa*	331558	−0.0291 (0.0868)	0.3354
sdecula II	tdecula XXIII	tdecim XXIII	*O. villosa*	280344	0.0337 (0.0952)	0.3544
(d) *M. neotredecim* to *M. tredecassini*						
cassini III	tcassini XIX	ntdecim XIX	*O. villosa*	336989	−0.0376 (0.0733)	0.5124
cassini III	tcassini XXIII	ntdecim XXIII	*O. villosa*	247511	0.0452 (0.0793)	0.5702
cassini II	tcassini XIX	ntdecim XIX	*O. villosa*	340239	−0.0459 (0.0752)	0.6101
cassini II	tcassini XXIII	ntdecim XXIII	*O. villosa*	253182	0.0279 (0.0833)	0.3353
(e) *M. neotredecim* to *M. tredecula*						
sdecula III	tdecula XIX	ntdecim XIX	*O. villosa*	344258	−0.0097 (0.0849)	0.1137
sdecula III	tdecula XXIII	ntdecim XXIII	*O. villosa*	248951	0.055 (0.0983)	0.5589
sdecula II	tdecula XIX	ntdecim XIX	*O. villosa*	331759	−0.038 (0.0899)	0.4224
sdecula II	tdecula XXIII	ntdecim XXIII	*O. villosa*	247764	−0.0014 (0.1003)	0.0139
(f) *M. tredecassini* to *M. tredecula*						
sdecula III	tdecula XIX	tcassini XIX	sdecim II,III	11517	−0.0104 (0.1468)	0.0706
sdecula III	tdecula XXIII	tcassini XXIII	sdecim II,III	9905	0.005 (0.1595)	0.0312
sdecula II	tdecula XIX	tcassini XIX	sdecim II,III	11166	−0.0107 (0.1495)	0.0713
sdecula II	tdecula XXIII	tcassini XXIII	sdecim II,III	9790	−0.0083 (0.1654)	0.0501
(g) *M. tredecula* to *M. tredecassini*						
cassini III	tcassini XIX	tdecula XIX	sdecim II,III	10819	0.0149 (0.1303)	0.1143
cassini III	tcassini XXIII	tdecula XXIII	sdecim II,III	9501	−0.0468 (0.1441)	0.3247
cassini II	tcassini XIX	tdecula XIX	sdecim II,III	11101	0.1041 (0.135)	0.7717
cassini II	tcassini XXIII	tdecula XXIII	sdecim II,III	9780	0.0105 (0.1447)	0.0724

P1, P2, P3 and O are operational taxonomic units (OTUs), in which P3 is the donor and P2 the recipient of introgression. The relationships among the OTUs are (((P1,P2),P3),O). Seven scenarios (a–g) were tested. Species: tdecim = *M. tredecim*; ntdecim = *M. neotredecim*; sdecim = *M. septendecim*; tcassini = *M*. *tredecassini*; cassini = *M. cassini*; tdecula = *M. tredecula*; sdecula = *M. septendecula*; *O. villosa* = *Okanagana villosa*

^a^ STD is the standard deviation of *D* obtained by the bootstrap procedure

^b^ The maximum *Z* score is 0.8193, which corresponds to *P* = 0.3579 (two-sided probability). Thus, no *D* values are significantly different from zero

### Demographic histories within species groups

To further investigate the historical process of life-cycle divergence, we inferred the demographic histories of broods within species groups using the program fastsimcoal2^27^, which analyses the joint site frequency spectra of synonymous SNPs. We used only high-quality SNPs from loci for which we could reliably infer reading frames. We considered three alternative scenarios of the relationships among broods (scenarios S1–S3), which reflected the possible diversification of the broods (Supplementary Fig. 1). In addition, we included three alternative models with gene flow between broods under each scenario, because recent divergence alone may not explain the low nodal support on the brood phylogenies. The three models were no gene flow, all possible recent and past gene flow (between ancestral populations and between current populations), and possible recent gene flow (between current populations). Thus, a total of nine models were compared in each of three species groups (Supplementary Fig. 1). For the Decim group, we included only samples of *M. septendecim* and *M. neotredecim* because *M. tredecim* had clearly diverged from the two species and gene flow between *M. tredecim* and parapatric *M. neotredecim* is virtually absent as was shown in our previous study^20^ and the ABBA-BABA test in the previous section.

We selected the best models of brood diversification based on model comparison using Akaike information criterion (AIC) weights and bootstrap proportions (Table 3). In all species groups, models with recent gene flow exhibited better fit than did models with no gene flow and those with both past and recent gene flow (Table 3). The best-fit scenarios were monophyly of both life cycles in the Decim and Cassini groups and monophyly of the 13-year species in the Decula group (Table 3, Fig. 4b). Note that the likelihood difference between recent gene flow models and past/recent gene flow models was marginal; the former models were favoured in AIC-based model comparisons because they had fewer parameters.

**Table 3:** Comparison of demographic models for the divergence of broods in the three species groups

Divergence scenario and gene flow pattern	No. of parameters	Log likelihood	AIC	ΔAIC	AIC weight	Bootstrap proportion of the model
Decim (*M. neotredecim* and *M. septendecim*)						
S1: (II, III), (XIX, XXIII)						
(1) No gene flow	9	−9159.70	18,337.398	55.89	5.7E-13	0.00
(2) Past/Recent	19	−9129.30	18,296.592	15.08	0.0004	0.01
**(3) Recent only**	**15**	**−9125.76**	**18,281.512**	**0.00**	**0.7806**	**0.92**
S2: (II, (XXIII, (III, XIX)))						
(1) No gene flow	9	−9211.42	18,440.838	159.33	2.0E-35	0.00
(2) Past/Recent	19	−9128.76	18,295.514	14.00	0.0007	0.00
(3) Recent only	15	−9129.98	18,289.966	8.45	0.0114	0.05
S3: (II, (III, (XIX, XXIII)))						
(1) No gene flow	9	−9150.28	18,318.564	37.05	7.0E-09	0.00
(2) Past/Recent	19	−9127.59	18,293.174	11.66	0.0023	0.00
(3) Recent only	15	−9127.10	18,284.19	2.68	0.2046	0.02
Cassini (*M. tredecassini* and *M. cassini*)						
S1: (II, III), (XIX, XXIII)						
(1) No gene flow	9	−7977.40	15,972.792	360.83	2.7E-79	0.08
(2) Past/Recent	19	−7789.05	15,616.092	4.13	0.0770	0.02
**(3) Recent only**	**15**	**−7790.98**	**15,611.962**	**0.00**	**0.6068**	**0.75**
S2: (II, (XXIII, (III, XIX)))						
(1) No gene flow	9	−8199.22	16,416.44	804.48	1.2E-175	0.01
(2) Past/Recent	19	−7792.81	15,623.612	11.65	0.0018	0.00
(3) Recent only	15	−7792.62	15,615.234	3.27	0.1182	0.08
S3: (II, (III, (XIX, XXIII)))						
(1) No gene flow	9	−7849.35	15,716.70	104.73	1.1E-23	0.03
(2) Past/Recent	19	−7792.77	15,623.538	11.58	0.0019	0.00
(3) Recent only	15	−7792.12	15,614.238	2.28	0.1944	0.03
Decula (*M. tredecula* and *M. septendecula*)						
S1: (II, III), (XIX, XXIII)						
(1) No gene flow	9	−6272.00	12,562.006	1095.14	5.9065E-239	0.00
(2) Past/Recent	19	−5717.92	11,473.838	6.97	0.0116	0.00
(3) Recent only	15	−5718.67	11,467.338	0.47	0.2989	0.32
S2: (II, (XXIII, (III, XIX)))						
(1) No gene flow	9	−6208.73	12,435.464	968.60	1.7765E-211	0.00
(2) Past/Recent	19	−5718.51	11,475.01	8.14	0.0064	0.00
(3) Recent only	15	−5718.66	11,467.314	0.45	0.3025	0.22
S3: (II, (III, (XIX, XXIII)))						
(1) No gene flow	9	−6098.49	12,214.98	748.11	1.3399E-163	0.00
(2) Past/Recent	19	−5719.41	11,476.816	9.95	0.0026	0.00
**(3) Recent only**	**15**	**−5718.43**	**11,466.868**	**0.00**	**0.3780**	**0.46**

The best fit model among nine models (three divergence scenarios and three patterns of gene flow; Supplementary Fig. 1) was selected using the AIC method. Bold letters indicate the best fit models with smallest AIC

*AIC* Akaike Information Criterion, Δ*AIC* AIC difference from the best model

The estimated divergence times of 13- and 17-year life cycles in the three groups were 197, 121 and 95 ky ago (kya) in the Decim, Cassini and Decula groups, respectively (Fig. 4b, Supplementary Table 1). These three divergences occurred between the Illinoian glacial period and the last glacial period. These divergence times are comparable to the times of the most recent common ancestor (tMRCA) for 13- and 17-year species pairs estimated in the maximum-likelihood tree, 213, 131 and 111 kya for the Decim, Cassini and Decula groups, respectively (Fig. 4b). The most recent common ancestor for 13-year cicada broods occurred 74, 64 and 17 kya in the Decim, Cassini and Decula groups, respectively (Supplementary Table 1). Thus, the split of the two major 13-year broods likely occurred during the last glacial period (Fig. 4b).

The estimated effective population size (*N*_e_) was consistent with the known biology of *Magicicada* (Fig. 4b, Supplementary Table 1). In the Cassini and Decula groups, *N*_e_ was larger in 13-year broods than in 17-year broods, which generally reflects the widespread range of 13-year species in these groups. By contrast, in the Decim group containing *M. septendecim* and *M. neotredecim*, *N*_e_ of the 17-year broods (*M. septendecim*) was larger than that of the 13-year broods (*M. neotredecim*), which suggests a recent origin for the narrowly distributed 13-year species *M. neotredecim*^4^. The current population sizes of 13-year and 17-year cicadas in each species group were larger than ancestral population sizes (except 17-year broods in the Decula group), which suggests recent population expansion associated with divergence of broods.

Estimated gene flow (*N*_e_*M*) between broods with the best models ranged from 0.01 to 23.8 migrants (individuals) per generation (Fig. 4c, Supplementary Table 1). For brood pairs in 13- or 17-year cicadas, gene flow was small between 17-year broods II and III, which are geographically separated, but 13-year brood pairs XIX and XXIII, which share lengthy boundaries, showed substantial gene flow (>1.0)^28^ in all species groups. For brood pairs between the 13- and 17-year cicadas, a substantial amount of gene flow (>1.0) was estimated to have occurred between all pairs in the Decim and Decula groups and between two of the four pairs in the Cassini group. Although the *N*_e_*M* confidence intervals were wide in each instance, the lower confidence limits of the gene flow between adjacent broods III and XIX were higher than 1.0 in the Decim and Decula groups, as well as between broods III and XXIII in the Decim group. In the Decula group, the *N*_e_*M* between broods II and XIX was greater than 1.0 despite the geographic separation of the samples, which indicates gene flow between eastern and western populations of brood XIX. In the Cassini group, the lower confidence limits of the gene flow between 13- and 17-year broods were low but non-zero (>0.003; Supplementary Table 1).

### Genomic divergence between 13-year and 17-year cicadas

**Fig. 5:**
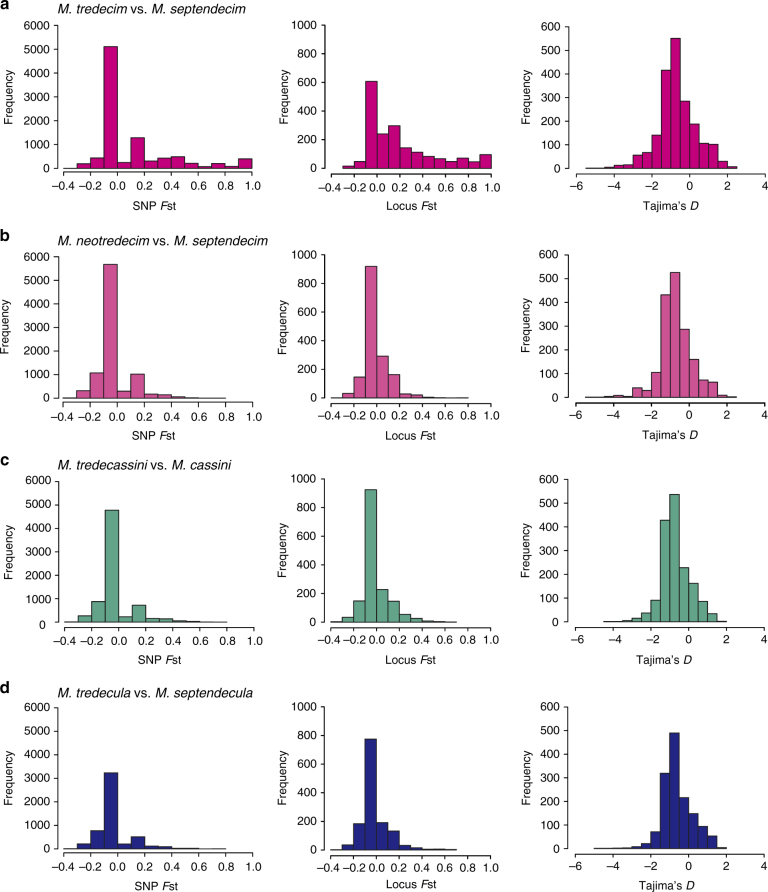
Genomic sequence divergence between 13- and 17-year species within species groups. Frequency distributions of *F*_st_ of individual SNPs (left), *F*_st_ of loci (middle) and Tajima’s *D* (right) are shown for *M. tredecim* vs. *M. septendecim* (**a**), *M. neotredecim* vs. *M. septendecim* (**b**), *M. tredecassini* vs. *M. cassini* (**c**), and *M. tredecula* vs. *M. septendecula* (**d**). Median and mean values for SNP *F*_st_: **a** median = 0.000, mean = 0.135; **b** median = 0.000, mean = −0.007; **c** median = 0.000, mean = −0.002; **d** median = 0.000, mean = −0.007; for locus *F*_st_: **a** median = 0.1111, mean = 0.2146; **b** median = 0.0000, mean = −0.0012; **c** median = 0.0000, mean = 0.0055; **d** median = 0.0000, mean = −0.0049; for Tajima’s *D*: **a** median = −0.7995, mean = −0.6886; **b** median = −0.8015, mean = −0.7065; **c** median = −0.8015, mean = −0.7046; **d** median = −0.7806, mean = −0.5952

We measured genomic divergence between four 13- and 17-year species pairs of three groups (the three pairs detailed above and the *M. tredecim*/*M. septendecim* pair) using the fixation index *F*_st_ for individual SNPs and loci (Fig. 5). In general, *F*_st_ did not indicate divergence between 17- and 13-year species except in the anciently diverged pair, *M. tredecim* and *M. septendecim* (Fig. 5). At the locus level, Tajima’s *D* values were generally negative, and only 16–22% of loci exhibited positive values (Fig. 5), which indicates that the loci were mainly under purifying selection, although population size expansion (Fig. 4b) may have also affected Tajima’s *D*. We also calculated *d*_*xy*_ between 13- and 17-year species as an absolute measure of nucleotide divergence, but the values of *d*_*xy*_ were strongly correlated with *π* (Supplementary Fig. 2) and did not capture the divergence between species. Therefore, we used only *F*_st_ for the analyses of shared outliers.

**Fig. 6:**
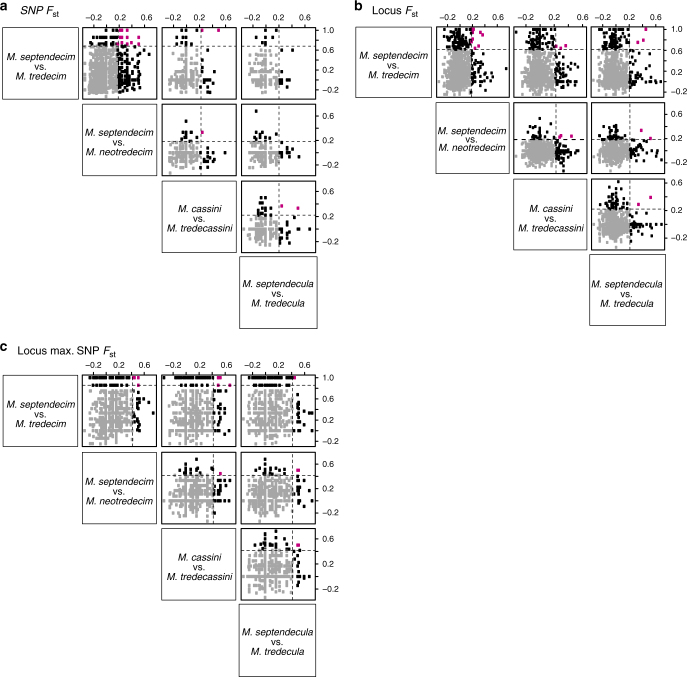
Pairwise comparisons of sequence divergence between two pairs of 13- and 17-year species to identify shared divergent SNPs and loci between groups. Comparisons of *F*_st_ of SNPs (**a**), Weir−Cockerham weighted *F*_st_ of loci (**b**), and maximum *F*_st_ among SNPs within loci (**c**). Dashed lines indicate 95% quantile of simulated neutral distribution of *F*_st_ values, which we defined as outliers. Closed red dots are outliers shared between species groups. Open black and grey dots show non-shared outliers and non-outliers, respectively. The observed number of shared outliers (*N*_shared_) and the probability (*P*) that observed numbers of shared outliers are obtained randomly for each of (**a**–**c**) are as follows (from left to right, and top to bottom). **a** *N*_shared_ = 25, *P* = 0.003; *N*_shared_ = 2, *P* = 0.81; *N*_shared_ = 0, *P* = 1; (row 2) *N*_shared_ = 1, *P* = 0.99; *N*_shared_ = 0, *P* = 0.99; (row 3) *N*_shared_ = 2, *P* = 0.81; **b** (row 1) *N*_shared_ = 9, *P* = 0.18; *N*_shared_ = 3, *P* = 0.99; *N*_shared_ = 3, *P* = 0.90; (row 2) *N*_shared_ = 3, *P* = 0.34; *N*_shared_ = 2, *P* = 0.31; (row 3) *N*_shared_ = 2, *P* = 0.43; **c** (row 1) *N*_shared_ = 6, *P* = 0.10; *N*_shared_ = 5, *P* = 0.31; *N*_shared_ = 1, *P* = 0.84; (row 2) *N*_shared_ = 1, *P* = 0.11; *N*_shared_ = 2, *P* = 0.004; (row 3) *N*_shared_ = 2, *P* = 0.01

One possible mechanism of parallel life-cycle divergence is parallel divergence at the same nucleotide sites or loci, which may be accompanied by divergence of linked genomic portions. Therefore, we searched for divergent SNPs or loci shared between the four pairs of 13- and 17-year species (six comparisons) using *F*_st_ values for each 13- and 17-year species pair (Fig. 6). We defined elevated *F*_st_ by simulating SNPs under the best demographic models inferred in the previous section and taking the 95% quantiles of simulated *F*_st_. Among 23,524 SNPs examined, 30 SNPs (0.1%) with elevated *F*_st_ (hereafter divergent SNPs) were shared by two species pairs (Fig. 6a, Supplementary Data 3), of which 27 SNPs were found in the within-Decim group comparison (i.e., *M. tredecim*/*M. septendecim* vs. *M. neotredecim*/*M. septendecim*). The other five comparisons yielded few or no shared divergent SNPs (Fig. 6a). The proportion of non-synonymous changes in the shared divergent SNPs was 0.47 (14/30) and did not significantly differ from genome wide proportion, 0.32 (*P* *=* 0.11, binomial test). At the locus level, we found 21 ‘divergent loci’ (0.7% of 2636 loci) with elevated *F*_st_ (Weir−Cockerham weighted *F*_st_)^29^, which were shared by two or more species pairs (Fig. 6b, Supplementary Data 3). Further, we selected the maximum SNP *F*_st_ for each locus as an alternative measure of locus-level divergence. We discovered 15 divergent loci (0.6%) with elevated maximum *F*_st_ shared by two or three pairs (Fig. 6c, Supplementary Data 3). In the only divergent locus shared by three pairs (the exception being the Decula pair), the SNPs with maximum *F*_st_ were located in different positions among the three pairs.

The above three analyses suggest that parallel genomic divergence associated with life-cycle divergence is uncommon. To clarify this, we conducted a permutation test to estimate the probability that the number of shared divergent SNPs or loci observed in each comparison were obtained by chance alone^22^. We found non-random occurrence of outliers of that number only for the within-Decim comparison in the SNP *F*_st_, none in the locus *F*_st_, and only two comparisons involving the Decula or Cassini groups and *M. neotredecim*/*M. septendecim* in the locus maximum *F*_st_ (the number of shared divergent SNPs or loci, *N*_shared_, and the permutational *P* values are given in the legend of Fig. 6a–c).

In the above outlier analyses, we obtained a total of 45 loci that exhibited elevated *F*_st_ at SNP or locus level between pairs of 13- and 17-year species (Supplementary Data 4). The functional annotation of these genes did not indicate enrichment of any kind of gene function. The 2636 loci studied included 21 genes involved in pathways potentially related to life cycle control (circadian clock, insulin signalling, insect hormone biosynthesis, MAPK signalling, and phototransduction^30–33^; Supplementary Data 5). However, the genes involved in these pathways were not found in the shared divergent loci.

## Discussion

Our phylogenetic analysis using mRNA sequences clearly resolved the branching pattern of the four major periodical cicada clades, consistent with those of previous studies that used mitochondrial and genome-wide (restriction-site-associated DNA; RAD) markers^6,20^. However, neither mRNA and RAD sequence data resolved the relationships among broods within species groups despite the vast amounts of data, whereas the mitochondrial gene tree partly resolved phylogeographic (eastern, middle and western) patterns within species groups^6^. However, phylogenetic and population genetic inferences with mitochondrial gene sequences can be distorted by introgressive hybridisation and incomplete lineage sorting of ancestral polymorphism^34^. Therefore, it was necessary to revisit the results of our previous study based mainly on mitochondrial data, especially to confirm the divergence process of broods with different life cycles.

Our demographic inference using a site frequency-based method provides new insights into the parallel divergence process of 13- and 17-year life cycles, revealing that the three species groups have nearly parallel demographic histories, with 13-year broods monophyletically diverged from 17-year broods in each species group. The Decim and Cassini groups share a common diversification pattern in which 13- and 17-year groups diversified first, whereas the Decula group had a slightly different history, as the 13-year group was derived from brood III (representing the western 17-year brood). However, the divergence time of the 13-year group from brood III is close to that of broods II and III in the Decula group; thus the differences in divergence patterns among the species groups may not be substantial. The present results differ from those found through mitochondrial phylogenetics^6^, in which 13-year broods in both the Decim and Cassini groups were found to have been derived from the western haplotype group, including brood III, whereas the origin of 13-year broods appears to have been polyphyletic in the Decula group. Our demographic inference also suggests population expansions following brood splits in each species group. This finding is consistent with the previous results using mitochondrial data that population expansions occurred after the last glacial period in the Decim and Cassini groups^6^ although the present study does not restrict the timing of population expansion to the post LGM except for Decula 13-year broods.

We estimated that life cycle divergence (i.e., the split between 13- and 17-year species) in the Decim group (excluding *M. tredecim*) occurred at the beginning of the Illinoian glacial period (200–130 kya), and those of the Cassini and Decula groups during the Sangamon interglacial period (130–115 kya) or early in the last (Wisconsin) glacial period (115–12 kya). Although the confidence intervals for these estimated times are wide (between 247 and 19 ky overall), the present estimates are much older than the divergence times estimated using mitochondrial gene sequences in our previous study^6^, suggesting divergence within the last 23 kya (i.e. after the last glacial maximum). Because estimated tMRCA for the species groups are similar between this study and our previous study (see Fig. 3 and Supplementary Table 2), the short divergence times revealed by mitochondrial data may reflect recent mitochondrial introgression between geographically adjacent broods. If our new inferences are correct, it follows that both life cycles have persisted in all species groups since at least the beginning of the last glacial period.

Notably, our demographic inference showed that gene flow has occurred between the 13- and 17-year species in each species group, particularly between species with geographically adjacent 13- and 17-year broods. Although it is difficult to discriminate ‘diverged populations with gene flow’ from ‘recently diverged populations with no gene flow after the divergence,’ our model comparison showed that models with no gene flow had the lowest likelihoods compared to other models with gene flow (Table 2). In addition, the SVDquartets tree (Fig. 4a) showed low resolution for brood relationships; this lack of resolution makes sense because SVDquartets is designed to accommodate cases where gene flow is absent and incomplete lineage sorting is the source of gene tree incongruence^35^.

Gene flow between neighbouring populations of 13- and 17-year broods (species) may have occurred in the year of their co-emergence, every 221 (13×17) years or during occasional off-schedule emergences of smaller number of individuals (called ‘stragglers’^36^). The 13- and 17-year cicadas within species groups do not show clear morphological or behavioural differentiation^4,5^; hence they could potentially hybridise^7,37^. The finding of gene flow between sister 13- and 17-year species may be odd, because historical records indicate stability of the boundary between 13- and 17-year broods^38^. It is possible that the synchronised life cycle among individuals of each brood has been strongly selected, and thus is stable in the face of occasional gene flow^9^.

We initially hypothesised that the difference between the two life cycles was controlled by a locus that regulates juvenile development and an ancestral polymorphism at the locus may have caused the parallel life cycle divergence through collateral genetic evolution. Our comparison of orthologous gene sequences between 13- and 17-year species, however, has not provided any substantial clues resolving the genetic basis of life cycle divergence. We searched for shared SNPs or diverged loci among the four pairs of 13- and 17-year species (i.e., including *M. tredecim*), which may be related to the regulation of life cycles. Such shared SNPs/loci would show elevated *F*_st_ and deep divergence if ancestral polymorphisms were responsible for cycle shifts; alternatively, shared SNPs/loci with shallow divergence would be detected if independent mutations were responsible. However, we found no divergent SNPs or loci that were shared by all pairs. Even if life cycle loci exist, they may be undetectable in reduced representation sequences such as the RNAseq used in this study, likely because the responsible regions are small regardless of whether they are ancestral polymorphisms or independent mutations. We also found that non-random parallel genomic divergence (in terms of *F*_st_) has not occurred among the four pairs of 13- and 17-year species, which may be expected in the parallel evolution of alternative phenotypes in different lineages^22,23^. If life cycle is controlled at multiple genetic levels rather than by a single mutation or a single diverged locus, any mutation in a group of genes within the same pathway could trigger a life cycle shift^17^. However, the results of functional annotation for the divergent loci between 13- and 17-year species showed no evidence of enrichment for a particular pathway or gene function. Thus, we have no conclusive information on the genetic control of life cycles at present.

Considering that we did not observe definitive genomic differences between the two life cycles, a non-genetic explanation for life cycle differences based on life cycle plasticity may not be ruled out completely. In a non-genetic scenario, different life cycles may be maintained by a threshold response of nymphs to clinal climatic factors such as the cumulative temperature during growing seasons. In fact, the geographic life cycle boundary (Fig. 1b) is predictable by local temperature data^39^. However, such an environmentally cued life cycle control may be unstable under fluctuating climatic conditions. In either case (i.e., genetic or non-genetic control of life cycle regulation), it would be necessary to conduct a thorough comparison of the whole genomic sequences between closely related 13- and 17-year species to fully explore the nature of life-cycle divergence in periodical cicadas.

## Methods

### RNA preparation and sequencing

We sampled 28 individuals from the seven known species of *Magicicada* (Supplementary Data 1). Four 13-year species were sampled from brood XIX (2011) and XXIII (2015), and three 17-year species from brood II (2013) and III (2014) during their emergences (Fig. 2a, Supplementary Data 1). Total RNA was extracted from head tissues using QIAGEN RNeasy. Libraries for sequencing were constructed and sequenced using the Illumina Hiseq2000 platform. Quality-filtered raw reads were deposited at the DNA Data Bank of Japan (DDBJ), in the DDBJ Read Archive (DRA).

### De novo assembly and SNP calling

The quality-filtered sequence reads were de novo assembled using the Trinity assembler version r20140717^40^ with the default parameter settings. Samples from the species groups (Decim, Cassini and Decula) were pooled, and consensus contigs of species groups were assembled. Within the Decim group, *M. tredecim* samples were separately assembled because *M. tredecim* is clearly diverged from the monophyletic group that includes *M. septendecim* and *M. neotredecim*^6,20^. Thus, we obtained consensus assemblies for *M. tredecim* and the remaining Decim (*M. septendecim/M. neotredecim*), Cassini (*M. cassini/M. tredecassni*), and Decula (*M. septendecula*/*M. tredecula*) species.

SNPs for each sample were called as follows. Reads of samples were mapped to the consensus contigs using bowtie2 version 4.1.2^41^, and variants were called with the ‘mpileup’ command in SAMtools version 1.2.0 and the ‘call’ command in BCFtools version 1.2.0^42^, which implements the likelihood method for multi-sample SNP calling. Only SNPs supported with coverage of ≥3 and a quality score ≥20 were retained. These SNPs were inserted into the contigs using the BCFtools ‘consensus’ command, with heterozygous sites retained using IUPAC-style ambiguity coding. Bases with coverage <3 were masked with N, and terminal Ns were removed. Contigs shorter than 300 bp were filtered out, and the longest isoform for each trinity sequence cluster was selected for downstream orthology clustering.

*Okanagana villosa* was selected as the outgroup species for clustering; this is the closest species available in the NCBI database. Contigs of the *O. villosa* transcriptome^43^ were downloaded from the Transcriptome Shotgun Assembly database (Accession: GAWQ02000001–GAWQ02051314) and filtered with the same criteria as used for the *Magicicada* trinity contigs; only contigs longer than 300 bp and the longest isoforms were retained for the following clustering.

### Orthology clustering

The consensus contigs of the samples were clustered into putative orthologous groups (loci) following the approach of Yang and Smith^44^. In brief, all-by-all BLASTN^45^ searches were conducted on all pairs of coding sequences of contigs, and then sequences with high similarity scores (evalue <1×10^–5^ and sequence identity >50%) were then clustered using MCL^46^. Then these homologous sequence clusters were aligned using MAFFT version 7.123^47^, and initial homologous trees were built using RAxML version 8.2.4^48^. Orthologous clusters were obtained following the ‘monophyletic outgroup’ criterion^44^, i.e. keeping the largest subtree that consisted exclusively of ingroup samples without duplication and monophyletic outgroup samples. Clustering was conducted using the phylogenomic dataset construction scripts available at https://bitbucket.org/yangya/phylogenomic_dataset_construction. To obtain the final alignments, consensus contigs were replaced by contigs with SNPs, and the sequences were realigned using PRANK version 14003^49^ using the default parameters. We retained orthologous clusters containing ≥27 *Magicicada* samples (>95% of samples) as a final data set. Clusters with overall genetic variation greater than 0.05 were removed as putative erroneous clusters. The longest cluster sequences were used for BLAST searches in the RefSeq protein database (see Supplementary Data 2 for annotated clusters).

### Phylogenetic inference

The maximum-likelihood (ML) phylogeny of individual samples was estimated using RAxML version 8.2.4^48^ with the concatenated alignment. RAxML was run using the ‘rapid bootstrap analysis and search for best-scoring ML tree’ algorithm with a GTR-Γ model and 100 bootstrap replicates. To estimate divergence time, the ML tree was converted to an ultrametric tree using LSD version 0.3beta^50^, with a calibration time of 3.89 mya at the node of the most recent common ancestor of all *Magicicada*^6^. Confidence intervals of node ages were obtained by 1000 bootstrap analysis. To account for the uncertainty for the time of the *Magicicada* MRCA, we also estimated divergence times with the calibration times of 3.08 and 4.69 mya, which were the lower and upper values of the 95% highest probability density interval. For each node, the confidence interval was determined as the oldest and youngest ages of 95% confidence intervals obtained from 1000 bootstrap replicates. A brood-level population tree was constructed using SVDquartets^24^ implemented in PAUP* version 4.0a147^51^. All clusters were concatenated, and SVDquartets was run using the ‘species tree’ option with 100 bootstrap replicates.

### ABBA-BABA test

We used the ABBA-BABA test with the *D*-statistic^25,26^ to test whether introgressive hybridisation has occurred between different 13-year species from different species groups or between distinct lineages of the same species group (i.e., *M. tredecim* vs. *M. neotredecim* in the Decim group). Under the assumption that population P1 and P2 are derived from population P3 and outgroup O, the ABBA-BABA test searches for evidence of hybridisation between P3 and P1 or P2 by comparing the frequencies of the site patterns ABBA and BABA. We set 17- and 13-year broods in the same species group as P1 and P2, respectively, and set one of 13-year broods from different species group as P3. An outgroup (O) was chosen from the closest available outgroup taxa. We tested the hybridisation of seven pairs of 13-year species with all four combinations of broods, totalling 28 comparisons (Table 1). *D*-statistics were calculated by a modified version of PyRAD version 3.0.66^52^, which accepts a fasta alignment as an input. The standard deviation of the *D*-statistic was obtained by a bootstrap resampling with 1000 replications.

### Demographic inference and model selection

We conducted demographic inference and model comparison using a method based on the site frequency spectrum (SFS) implemented in fastsimcoal2 version 2.5.2.21^27^. Synonymous SNPs were selected from the alignments of clusters, and folded joint SFSs of four populations representing 17-year broods II and III and 13-year broods XIX and XXIII were obtained with minimum site frequencies (5%) using Arlequin version 3.5^53^. Then the likelihoods of demographic scenarios were calculated using fastsimcoal2. Monomorphic sites were excluded from the likelihood calculations with the ‘removeZeroSFS’ option because we could not estimate the accurate number of monomorphic sites for synonymous SNPs. According to this option, the effective population size (*N*_e_) of one population (brood XIX for Decim, brood II for Decula and Cassini) was fixed to the value calculated from the average genetic variation (*π*) of the population and the relationship, *π* = 4*N*_e_*μ*, where *μ* is the *Magicicada*-specific mutation rate estimated as below.

We estimated the mean mutation rate from the present mRNA sequence data using the previously estimated age of several major nodes in the *Magicicada* phylogenetic tree^6^ and the node heights of the ML tree as estimated from the present mRNA sequence data as described above, and assuming a time-dependent substitution rate^54^. We also assumed a generation time of 15 years, the average of 13 and 17 years. Based on the ML tree resulting from concatenated mRNA sequences, node heights for seven clades are obtained (Supplementary Table 2). Using the corresponding node ages and a generation time of 15 years, the substitution rate per site per generation at each node was calculated. The substitution rate decayed over time towards an asymptote, as predicted^54^. Then, using the R package ‘nls’, the substitution rate and node age data were fitted to a non-linear model with the time-dependent evolutionary rate equation^54^:

$${\mathrm{Rate}}\left( t \right) = \mu \;\exp \left( { - \lambda t} \right) + k,$$

where *µ* is the instantaneous mutation rate, and *λ* is inversely proportional to the half-life of the rate decay, and *k* is a finite asymptotic evolutionary rate. As a result, we obtained estimates of these variables as *µ* = 0.008494, *λ* = 2.9185 and *k* = 0.006849 (per site per million generations). At *t* = 0, the rate *µ* + *k* equals 0.0153 per million generations (=1.53×10^−8^ per generation). This value was used as the mutation rate in the demographic analysis.

We included the following three alternative scenarios in the model comparison, which are based on known phylogeographic trees and the two life cycles:

Scenario S1: 13- and 17-year broods form monophyletic groups ((II, III), (XIX, XXIII));

Scenario S2: geographically adjacent sampled broods form clades irrespective of their life cycles (II, (XXIII, (III, XIX)));

Scenario S3: 13-year species are monophyletic, and adjacent 17-year broods are closer to these (II, (III, (XIX, XXIII))).

To assess the effects of gene flow, we included three models of gene flow between broods under the three population divergence scenarios listed above. The three models were ‘no gene flow’; ‘past and recent gene flow’, where gene flow exists between all current and ancestral populations; and ‘recent gene flow only’, where gene flow only exists between current populations. In total, nine models were used in the model comparison (Supplementary Fig. 1).

We chose the best model using AIC values and AIC weights^55^ calculated from composite likelihoods of the models, as recommended by Excoffier et al.^27^. In addition to model comparison with maximum likelihood inference, we performed bootstrap resampling of 100 replicates with Poisson approximation^56^ and recorded the bootstrap proportions, i.e., the proportions of replicates for which a given model was repeatedly chosen as the best model^57^.

### Population genomic measures

To characterise the within- and between-species genetic profiles of 17- and 13-year *Magicicada* species, population genetic measures were calculated for each orthologous cluster (locus). Genetic variation (*π*) and the number of segregating sites (*S*) within seven species were calculated. Tajima’s *D*^58^ was calculated to detect purifying or balancing selection in each species group. As a measure of net divergence between 13- and 17-year species, *F*_st_^29^ was calculated for each SNP as an SNP-level measure of divergence and for each cluster as a locus-level measure of divergence using the R version 3.3.3^59^ package ‘pegas’^60^. We used a weighted average of *F*_st_ values in a locus as a locus-level estimator per the method of Weir and Cockerham^29^. Maximum *F*_st_ values within a locus were collected as an alternative measure of locus-level divergence. We also calculated the average number of pairwise differences, *d*_*xy*_, for each locus between 13- and 17-year species because this index is recommended as an absolute measure of population divergence^61^.

Due to the sparse number of SNPs within loci and small sample sizes within populations, we were not able to reliably phase the genotypes. Therefore, we employed the repeated random haplotype sampling (RRHS) strategy^62^ when phase information was required. RRHS randomly assigns one of two possible genotypes at heterozygous sites. Thus, *π*, *d*_*xy*_ and Tajima’s *D* were repeatedly calculated with 100 RRHS replicates, and their averages were used as estimates.

### Outlier analysis for diverged genomic portions

To detect diverged genomic portions associated with the divergence of 13- and 17-year species, we conducted outlier analyses of *F*_st_ for each SNP, *F*_st_ for each locus, and maximum *F*_st_ among all SNPs within each locus. *F*_st_ is an inappropriate measure of population differentiation when it is highly negatively correlated with nucleotide diversity^63^. However, in our case, *F*_st_ was not correlated with mean nucleotide diversity at the locus level except for a weak negative correlation in the *M. tredecim*/*M. septendecim* pair (Supplementary Fig. 2). Note that *F*_st_ and mean nucleotide diversity are expected to be uncorrelated with each other when demographic factors (e.g., gene flow, genetic drift) outweigh the effect of mutations, whereas a negative correlation is expected between these measures in the opposite situation^63^. Meanwhile, the nucleotide divergence *d*_*xy*_, which is considered a more appropriate measure of population differentiation^61^, was strongly positively correlated with nucleotide diversity and hence may lead to false discovery of elevated *d*_*xy*_ at regions with high nucleotide diversity^64^ (Supplementary Fig. 2). Thus, the use of *F*_st_, rather than *d*_*xy*_, was considered appropriate in the present case.

We defined the SNPs/loci with elevated *F*_st_ values as ‘divergent SNPs/loci’. To determine thresholds to define elevated *F*_st_, we simulated up to 10,000 unlinked SNPs for the best-fitting demographic models selected above using fastsimcoal2 and calculated *F*_st_ between 13- and 17-year broods. The 95% quantile of the simulated statistics was chosen as the threshold to define elevated *F*_st_. The divergent SNPs/loci shared by two or more comparisons between two pairs of 13- and 17-year species were considered as ‘shared divergent SNPs/loci’, which are the candidate SNPs/loci responsible for the parallel life cycle divergence. The threshold value for the maximum *F*_st_ for a locus was determined by repeatedly taking a maximum of five *F*_st_ values of simulated SNPs to generate a distribution of maximum of *F*_st_ and obtaining the 95% quantile of this distribution. To determine the threshold to define elevated locus-level *F*_st_, we simulated linked sites of 2500 bp long for 5000 times (replicates) under the same demographic model. The weighted average of *F*_st_ values for SNPs in the linked sites was calculated each time, and the 95% quantile of the 5000 average *F*_st_ values were chosen as the threshold.

The number of divergent SNPs or loci with elevated *F*_st_ shared by two or more comparisons (i.e., ‘shared divergent SNPs/loci’) was considered an indicator of parallel divergence. The statistical significance of the numbers of shared divergent SNPs or loci was tested with permutation tests with 1000 replicates, which estimated the probability that the number of shared divergent SNPs or loci observed in each comparison were obtained by chance alone. For the shared divergent loci, functional annotations were made using DAVID Bioinformatics Resources 6.8^65,66^.

### Data availability

The raw sequence reads used in the present study are available from the DDBJ Read Archive (DRA) of the DNA Data Bank of Japan (DDBJ) (BioProject, PRJDB4567; BioSample, SAMD00047121–SAMD0004712147148). Other relevant data and input files used in the fastsimcoal2 runs are available via Figshare at 10.6084/m9.figshare.c.4011520^67^.

## Electronic supplementary material

Supplementary Information(PDF 783 kb)

Description of Additional Supplementary Files(DOCX 12 kb)

Supplementary Data 1(XLSX 42 kb)

Supplementary Data 2(XLSX 426 kb)

Supplementary Data 3(XLSX 12 kb)

Supplementary Data 4(XLSX 32 kb)

Supplementary Data 5(XLSX 42 kb)
