# Supplementary material for: Triplicate parallel life cycle divergence despite gene flow in periodical cicadas
Source: Commun Biol. 2018 Apr 19;1:26. doi: 10.1038/s42003-018-0025-7 (PMC6123741; doi:10.1038/s42003-018-0025-7)
Supplement: Supplementary file 1 — Supplementary Information(PDF 783 kb) [file 42003_2018_25_MOESM1_ESM.pdf]

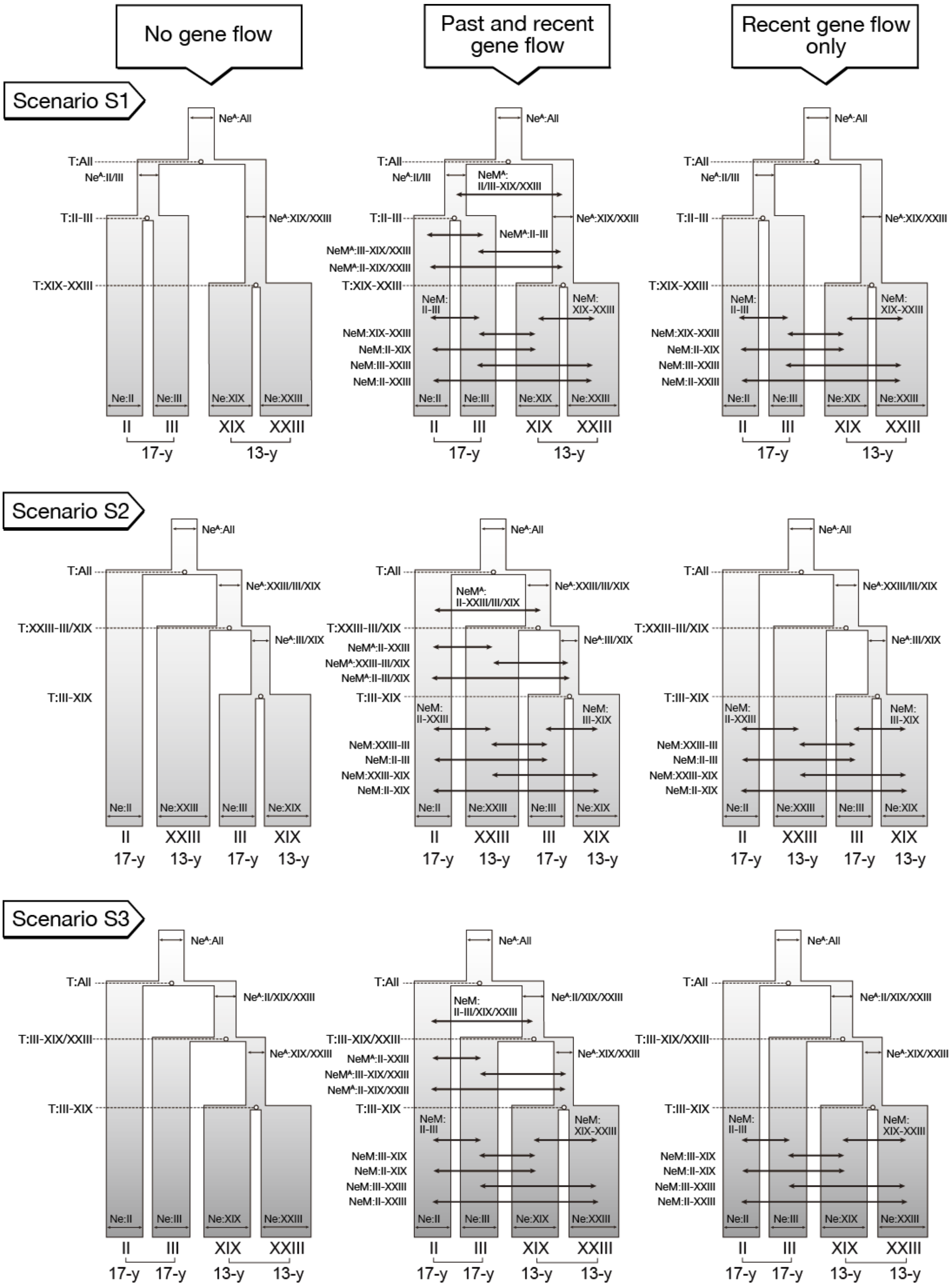

Supplementary Figure 1. Nine demographic hypotheses of divergence among four broods used in the simulation study with fastsimcoal2. Three branching patterns of broods (scenarios S1–S3) and three patterns of gene flow (no gene flow, past and recent gene flow, recent gene flow only) are considered.

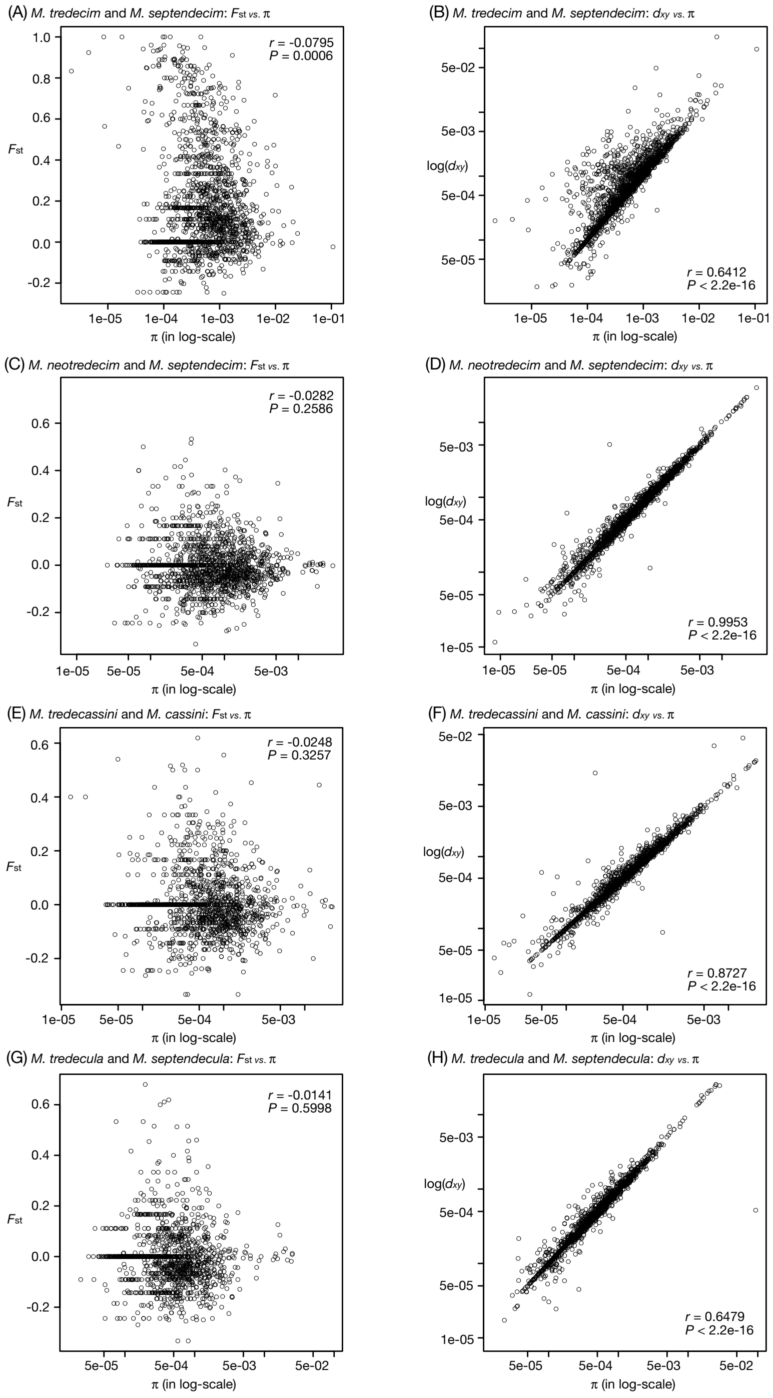

Supplementary Figure 2. Relationships between Weir & Cockerham weighted F_{st} and mean intraspecific nucleotide diversity (π) per locus (A, C, E, G), and between average interspecific nucleotide difference d_{xy} and mean intraspecific nucleotide diversity (π) per locus (B, D, F, H) for four pairs of 13- and 17-year species. d_{xy} and π are $\log_{10}$-transformed. The Pearson's product moment correlation (*r*) and *P*-value calculated using non-transformed values are given in each panel.

Supplementary Table 1. Estimated demographic parameter values and confidence intervals for the selected demographic model for each pair of 13- and 17-year species.

(A) Decim: best model= scenario S1/recent gene flow only

Parameter: brood	Maximum-likelihood estimate	95% confidence limit	
		Lower (2.5%)	Upper (97.5%)
Effective population size			
N_e:II	15785	9878	25699
N_e:III	13111	8854	23721
N_e:XIX	10961	NA	NA
N_e:XXIII	11003	7982	20630
N_eA:All	9681	6650	11143
N_eA:II/III	3219	1051	7158
N_eA:XIX/XXIII	2951	1236	12057
Divergence time (generations; years in parentheses)			
T:All	13147 (197205)	4238 (63574)	16461 (246915)
T:II-III	7792 (116880)	1648 (24722)	9507 (142602)
T:XIX-XXIII	4947 (74205)	928 (13919)	6586 (98796)
Migration rate (individuals/generation)			
N_eM:II-III	1.830	0.334	15.595
N_eM:II-XIX	6.557	0.005	9.314
N_eM:II-XXIII	13.059	0.044	15.441
N_eM:III-XIX	15.385	1.573	18.975
N_eM:III-XXIII	23.801	1.454	18.567
N_eM:XIX-XXIII	7.659	5.029	19.996

(B) Cassini: best model= scenario S1/recent gene flow only

Parameter: brood	Maximum-likelihood estimate	95% confidence limit	
		Lower (2.5%)	Upper (97.5%)
Effective population size			
N_e:II	8508	NA	NA
N_e:III	11420	5709	17060
N_e:XIX	13782	7195	19395
N_e:XXIII	14406	8496	26831
N_eA:All	8663	3261	9695
N_eA:II/III	1108	1079	7616
N_eA:XIX/XXIII	3435	1260	33299
Divergence time (generations; years in parentheses)			
T:All	8048 (120720)	1293 (19388)	12328 (184914)
T:II-III	5671 (85065)	991 (14867)	8088 (121325)
T:XIX-XXIII	4255 (63825)	375 (5622)	5899 (88480)
Migration rate (individuals/generation)			
N_eM:II-III	0.253	0.004	3.572
N_eM:II-XIX	0.023	0.003	4.447
N_eM:II-XXIII	3.061	0.010	4.356
N_eM:III-XIX	0.013	0.024	12.620
N_eM:III-XXIII	8.261	0.126	11.984
N_eM:XIX-XXIII	10.117	1.282	16.175

(C) Decula: best model= scenario S3/recent gene flow only

Parameter: brood	Maximum-likelihood estimate	95% confidence limit	
		Lower (2.5%)	Upper (97.5%)
Effective population size			
N_e:II	7331	NA	NA
N_e:III	7560	3794	17751
N_e:XIX	16916	9006	23437
N_e:XXIII	7556	2545	13611
N_e A:All	8618	3594	11880
N_e A:III/XIX/XXIII	7129	2575	10696
N_e A:XIX/XXIII	1812	1088	7555
Divergence time (generations; years in parentheses)			
T:All	7688 (115320)	2306 (34597)	18681 (280209)
T:III-XIX-XXIII	6331 (94965)	1432 (21483)	14425 (216372)
T:XIX-XXIII	1110 (16650)	362 (5423)	5650 (84746)
Migration rate (individuals/generation)			
N_eM:II-III	0.033	0.007	12.171
N_eM:II-XIX	13.571	5.499	17.347
N_eM:II-XXIII	1.365	0.005	8.648
N_eM:III-XIX	15.397	6.534	21.923
N_eM:III-XXIII	5.734	0.016	18.697
N_eM:XIX-XXIII	7.559	0.057	14.129

Parameters: N_e, effective population size (NA, fixed as reference); T, divergence time in generations (15 years/generation); N_eM, migration rate. Confidence intervals were obtained by bootstrap replications. Effective population sizes with NA for the confidence intervals were fixed as references.

Supplementary Table 2. The most recent common ancestor (MRCA) nodes, node heights in the maximum-likelihood tree, previously estimated node ages[1] and calculated substitution rates used to estimate the mutation rate.

MRCA node for clade:	Node height (ML tree)	Estimated age in million years	Substitution rate per site per generation
All *Magicicada*	0.00174938	3.89	6.7457×10^{9}
Cassini + Decula	0.00110075	2.51	6.5782×10^{9}
Decim	0.00037036	0.53	1.0482×10^{8}
Cassini	0.00015851	0.32	7.4303×10^{9}
Decula	0.00014580	0.23	9.5090×10^{9}
M. septendecim + *M. neotredecim*	0.00023147	0.27	1.2860×10^{8}
M. tredecim	0.00011704	0.13	1.3504×10^{8}

Supplementary References

1. Sota, T. *et al.* Independent divergence of 13-and 17-y life cycles among three periodical cicada lineages. *Proc. Natl. Acad. Sci. U. S. A.* **110,** 6919–6924 (2013).
