# Supplementary material for: Triplicate parallel life cycle divergence despite gene flow in periodical cicadas
Source: Commun Biol. 2018 Apr 19;1:26. doi: 10.1038/s42003-018-0025-7 (PMC6123741; doi:10.1038/s42003-018-0025-7)
Supplement: Supplementary file 2 — Description of Additional Supplementary Files(DOCX 12 kb) [file 42003_2018_25_MOESM2_ESM.docx]

**Description of Additional Supplementary Files**

File Name: Supplementary Data 1

Description: List of RNA sequence samples for *Magicada*.

File Name: Supplementary Data 2

Description: Results of blasting against Refseq database for 2636 orthologous clusters (gene loci) selected for the analyses.

File Name: Supplementary Data 3

Description: Diverged SNP or locus shared by two or more comparisons of 13- and 17-year species within species groups.

File Name: Supplementary Data 4

Description: Functional annotation of 45 non-duplicated outlier loci.

File Name: Supplementary Data 5

Description: Genes involved in pathways potentially related to life cycle control.
